# siMS score- method for quantification of metabolic syndrome, confirms co-founding factors of metabolic syndrome

**DOI:** 10.3389/fgene.2022.1041383

**Published:** 2023-01-04

**Authors:** V. Dimitrijevic-Sreckovic, H. Petrovic, D. Dobrosavljevic, E. Colak, N. Ivanovic, D. Gostiljac, S. Ilic, D. Nikolic, J. Gacic, I. Soldatovic

**Affiliations:** ^1^ University Clinical Center of Serbia, Clinic of Endocrinology, Diabetes and Metabolic Diseases, Belgrade, Serbia; ^2^ Faculty of Medicine, Belgrade University, Belgrade, Serbia; ^3^ Department of Clinical Genetics, University Children’s Hospital, Belgrade, Serbia; ^4^ Clinic of Dermatovenereology, Belgrade, Serbia; ^5^ Institute of Medical Biochemistry, Belgrade, Serbia; ^6^ Clinical Center Bezanijska Kosa, Belgrade, Serbia; ^7^ Institute for Medical Statistics and Informatics, Belgrade, Serbia

**Keywords:** siMS score, insulin resistance, metabolic syndrome, obesity, non-alcoholic fatty liver disease

## Abstract

**Background:** Adipose tissue is a dynamic endocrine organ, a highly active metabolic tissue, and an important source of cytokines. Inflammatory factors play an important role in visceral obesity associated with insulin resistance (IR), metabolic syndrome (MS), hypertension, non-alcoholic fatty liver disease (NAFLD), diabetes mellitus type 2 (DM2), endothelial dysfunction (ED) and atherosclerosis.

**Objectives:** To examine corelation of siMS score, as a quantification method for metabolic syndrome (MS), with insulin resistance, glucoregulation parameters, as with other co-founding factors of MS, inflammation and thrombosis factors, microalbuminuria, uric acid, fatty liver index (FLI) and homocysteine.

**Methods:** The study included 451 obese individuals with pre–metabolic syndrome (pre-MS) and MS (age 16–75, body mass index (BMI) > 25kg/m^2^) classified into two groups: I-age 10–30 (167 patients); II-age 31–75 (284 patients). International Diabetes Federation (IDF) classification was applied for diagnosing metabolic syndrome. Patients with less than three criteria indicated below were considered pre-metabolic syndrome. siMS risk score was used.

**Results:** siMS score increased with age: I-3.03 ± 0.87, II-3.27 ± 0.90. siMS score correlated with associated factors of MS: hyperinsulinemia and IR, ALT, gama-GT, FLI, uric acid in both groups and CRP (*p* < 0.01) in group I. Correlations in II group: siMS score with PAI-1 (*p* = 0.01), microalbuminuria (*p* = 0.006), homocysteine ​​(*p* = 0.076).

**Conclusion:** Correlation of siMS score with HOMA-IR confirmed that hyperinsulinism and insulin resistance are in the basis of MS. Correlation of siMS score with parameters of NAFLD, CRP, PAI-1, uric acid, microalbuminuria and homocysteine indicates that they are significant co-founding factors of MS. Correlation of siMS score with PAI-1, microalbuminuria, homocysteine, indicates higher risk for progression of endothelial dysfunction and atherosclerosis with age.

## Introduction

The obesity epidemic in the world is influenced by a diet rich in simple carbohydrates from sweets, pastries, white flour products, fats and proteins of animal origin and fried food. This trend is followed by insufficient intake of dietary fiber from fruits and vegetables, reduced use of olive oil and sea fish, as well as reduced physical activity. Mediterranean diet is an example of food rich in fruits and vegetables, sea fish, olive oil and represents diet of choice when it comes to overweight and obese patients in our practice. With increase in the degree of obesity, the risk for the development of metabolic syndrome (MS) rises, which promotes the development of diabetes mellitus type 2 (DM2), hyperlipoproteinemia, hypertension, cardiovascular disease, non-alcoholic fatty liver disease (NAFLD), infertility and cancer. Waist circumference is the simplest but most reliable indicator of the size of intra-abdominal fat depots (Pouliot et al., 1994). International Diabetes Federation (IDF) highlights abdominal obesity, emphasized by waist circumference (WC), as one of the main causes of insulin resistance (IR) (Alberti et al., 2005). Obesity is characterized by hypertrophy and hyperplasia of adipocytes, which can cause reduced blood supply and adipose tissue hypoxia. In a condition of adipose tissue hypoxia, necrosis, and infiltration of macrophages into the adipose tissue and excessive production of active metabolites such as proinflammatory cytokines [interleukin-6 (IL-6), tumor necrosis factor-alpha (TNF-α)] occur (Cinti et al., 2005). Cytokines from adipocytes and macrophages trigger an inflammatory response, cause vasoconstriction of blood vessels and the development of hypertension and IR, the main characteristic of MS. Insulin inhibits gluconeogenesis and glycogenolysis, increases glucose uptake in muscle and inhibits lipolysis in adipose tissue, failure of these three mechanisms is the basis of IR. Hyperinsulinism stimulates the secretion of endothelin-1 which causes proliferation of vascular smooth myocytes leading to vasospasm and further development of atherosclerosis. Proinflammatory cytokines TNF-α, IL-6 as other factors further promote IR and lipolysis of adipose tissue triglycerides by increasing the concentration of free fatty acids (FFA) in the blood and their compensatory uptake by the liver and hyperproduction of VLDL. This massive influx of FFA and cytokines lead towards NAFLD (Weisberg et al., 2003). The appearance of hyperinsulinism, reduced insulin sensitivity and IR can precede the appearance of MS in the following years if another factor is added and therapeutic measures are not taken (Dimitrijevic-Sreckovic, 2011a). NAFLD is characterized by the presence of biochemical parameters in the form of elevated alanine aminotransferase (ALT) and gamma-GT (GGT) values, and ultrasound finding of a fatty liver (Mauss et al., 2009). IR lies in the pathogenesis of NAFLD, therefore we emphasize the “two-hit theory” (Day and James, 1998). The first blow is the development of IR, and therefore the accumulation of lipids develops due to the increased influx of FFA into hepatocytes and the appearance of steatosis in the liver. The second hit leads to further hepatocyte damage, inflammation, and consequent fibrosis. The factors responsible for this second blow are oxidative stress, lipid peroxidation, proinflammatory cytokines and mitochondrial dysfunction. We highlight the role of visceral adipose tissue in the state of developed IR because it is precisely the source of FFA that reach the liver in high concentrations. Dysfunction at the level of hepatocyte mitochondria leads to further accumulation of lipids in the cells, leading to oxidative stress and the creation of a vicious circle, which represents the second blow (Wei et al., 2008). In that state of IR and massive influx of FFA into the liver, there is increased VLDL production causing hypertriglyceridemia and reduction of HDL-cholesterol. Cytokines and FFA increase the production of fibrinogen, plasminogen activator inhibitor- 1 (PAI-1) and C-reactive protein (CRP) by the liver, which together with the hyperproduction of PAI-1 by adipose tissue causes a prothrombogenic state. Obesity *per se,* with one or two risk factors (defined as pre-MS patients) and MS patients have increased insulin secretion, hyperinsulinism, IR, increased thrombotic factors PAI-1 and low antioxidant protection. Our earlier results showed a positive correlation of abdominal obesity with IR, body mass index (BMI), insulin secretion, as well as a negative correlation with BMI and glutathione peroxidase. This confirms that insulin secretion, hyperinsulinism and IR are in a mutual relationship with reduced antioxidant protection in obese patients (Dimitrijevic-Sreckovic et al., 2007). Previous studies have shown a correlation of CRP with risk factors for MS such as abdominal obesity, hyperinsulinemia, IR, hypertriglyceridemia, and low HDL-cholesterol (Officers and Coordinators for the ALLHAT Collaborative Research Group, 2002).

MS is defined by a forenamed cluster of risk factors as abdominal obesity, hyperglycemia, hypertension, hypertriglyceridemia, and low HDL-cholesterol, also there are other risk factors such as elevated uric acid and hyperhomocysteinemia. The main cause of increased uric acid is hyperinsulinemia, as well as reduced uric acid excretion in renal function disorders. Increased intake of fructose in the diet stands out as the main cause of elevated uric acid, risk for MS, diabetes, hypertension, and other comorbidities (Soltani et al., 2013). Elevated homocysteine ​​values ​​correlate with hyperinsulinism and IR, which leads to increased oxidative stress and oxidative damage to the vascular endothelium, endothelial function disorders, elevated blood pressure, and atherosclerosis (Sreckovic et al., 2017). Sertoglu et al. (2015) in their research point out that homocysteine ​​values ​​correlate with components of the metabolic syndrome, especially with systolic blood pressure. The association of homocysteine ​​with other factors of the metabolic syndrome represents a risk for atherosclerosis and cardiovascular diseases. Hyperhomocysteinemia and microalbuminuria associated with hyperinsulinemia and IR lead to endothelial damage and increased risk for atherosclerotic vascular complications (Meigs et al., 2001). Disturbances of glycoregulation, lipid status and hypertension are, along with abdominal obesity, the main factors of MS. Study by Alberti et al. (2009) have shown that the presence of MS increases the risk of developing diabetes mellitus type 2 (DM2) fivefold and the development of cardiovascular diseases twice, which has been the subject of numerous scientific studies in recent decades. The lack of an adequate way to quantify the metabolic syndrome represented a potential problem in the diagnosis and follow-up of these patients. Soldatović I. designed the siMS score as a method for quantifying the metabolic syndrome as well as the siMS risk score for determining the risk of cardiovascular and cerebrovascular events that were evaluated on 528 patients of the Center for Nutrition and Prevention of Metabolic Disorders, Clinic for Endocrinology, Diabetes and Metabolic Diseases, University Clinical Center of Serbia (Soldatovic et al., 2016).

## Aims of the study

To examine corelation of siMS score, as a quantification method for metabolic syndrome, with IR, glucoregulation parameters, as with other co-founding factors of MS, inflammation factor CRP and thrombosis factor PAI-1, uric acid, microalbuminuria, fatty liver index (FLI) and homocysteine.

The second goal of the work was to calculate the SiMS score in overweight and obese patients without glycoregulation disorders, patients with prediabetes (impaired fasting glucosae-IFG and impaired glucose tolerance-IGT) and newly diagnosed DM2 patients.

## Material and methods

The study included 451 obese individuals with pre–MS and MS (age 16–75, BMI > 25kg/m^2^ overweight and BMI > 30kg/m^2^ obese) classified into two groups: I-age 16–30 (167 patients); II-age 31–75 (284 patients). Anthropometric parameters measured were waist circumference (WC) and BMI. WC was measured with an inelastic tape at the midpoint between the top of the iliac bone and the lower edge of the ribs, in the horizontal plane and at the end of expiration. BMI was calculated as body weight in kilograms divided by the square of body height in meters. IDF classification was applied for diagnosing MS (Alberti et al., 2005). Patients with less than three criteria indicated below were considered pre-MS (Dimitrijevic-Sreckovic et al., 2007). International Diabetes Federation (IDF) criteria for diagnosing MS, lowered the values ​​for abdominal obesity in comparison to ATP III criteria. WC for the European population in men/women ≥ 94/≥ 80 cm; and the presence of two other factors: triglycerides ≥ 1.7 mmol/L or applied therapy; HDL cholesterol <1.03 mmol/L in men or <1.29 mmol/L in women or applied therapy; blood: systolic pressure (SBP)/diastolic pressure (DBP) ≥ 135/85 mmHg or use of antihypertensive therapy; blood glucose ≥5.6 mmol/L and/or previously diagnosed DM2 (Alberti et al., 2005).Values ​​of glycaemia and insulin were measured during an oral glucose tolerance test (OGTT) with 75 g of glucose at 0, 30 and 120 min. OGTT was used to evaluate the extent of glicoregulaation disorder: IFG and IGT as well as patients with the newly discovered DM2. Glycemia was determined by the enzymatic spectrophotometric method. Insulin was measured using the radioimmunoassay method. IR was determined by homeostatic model assessment (HOMA-IR) HOMA-IR = insulinemia (mU/l) x glycaemia (mmol/L)/22, 5. Lipid status was determined by total cholesterol, HDL-cholesterol, LDL-cholesterol, and triglycerides using standard spectrophotometer methods. HbA1C, as a parameter of long-term glicoregulation was determined by spectrophotometry. In this paper, the associated MS parameters were analyzed: IR, CRP, PAI-1, uric acid, liver, and renal function parameters. Serum CRP was measured by immunometric assay while PAI-1 was determined by plasminogen substrate essay. Homocysteine was determined on Abbott’s Architect analyzer, using CMIA technology. Uric acid was determined by standard laboratory test. Liver function parameters determined were aspartate aminotransferase (AST), alanine aminotransferase (ALT), g-glutamyl-transpeptidase (GGT). Fatty liver index (FLI) = [e 0.953*loge (triglycerides) + 0.139*BMI +0.718*loge (GGT) + 0.053*waist circumference−15.745)/(1 + e 0.953*loge (triglycerides) + 0.139*BMI +0.718*loge (GGT) + 0.053*waist circumference−15.745] * 100. Renal function parameters determined were urea, creatinine, creatinine clearance, microalbuminuria from 24-hour urine, using immune-nephelometric method.

Soldatovic I. created new siMS score as a simple score for clinical use, which in this paper is correlated with associated MS factors and other laboratory parameters (Soldatovic et al., 2016). The value of the siMS score was determined in the group of overweight, obese, IFG, IGT and newly diagnosed DM2.

The formula for siMS score using MS reference values is calculated as follows:

$$siMS\;score=\frac{2x\;Waist}{Height}+\frac{Gly}{5.6}+\frac{Tg}{1.7}+\frac{TA\;systolic}{130}-\frac{HDL}{1.03or1.3\left(male\;or\;female\right)}$$

### Statistical analysis

Results are presented as count (%), means ± standard deviation or median (25th-75th Percentile) depending on data type and distribution. Groups are compared using parametric (t test) and non-parametric (Chi-square, Fisher’s Exact test, Mann-Whitney U test) tests. To assess correlation between variables Pearson and Spearman correlation was used. All *p* values less than 0.05 were considered significant. All data were analyzed using SPSS 20.0 (IBM Corp. Released 2011. IBM SPSS Statistics for Windows, Version 20.0. Armonk, NY: IBM Corp.) and R 3.4.2 (R Core Team (2017). R: A language and environment for statistical computing. R Foundation for Statistical Computing, Vienna, Austria. URL https://www.R-project.org/).

## Results

Patients with pre-MS and MS had increased WC: (I-104.0 ± 17.7, II-104.6 ± 15.4 cm), BMI: (I-33.35 ± 7.07, II-33.18 ± 6.85 kg/m^2^), systolic pressure (I-124.8 ± 14.9, II-130.9-17.7 mmHg), diastolic pressure (I-81.6 ± 10.5, II-84.7-11.9 mmHg), mean insulinemia [I-55.47 (31.57–88.07), II-40.27 (26.5–60.53) U/ml], HOMA-IR [I- 4.9 (3.54–7.5), II-4.01 (2.99–6.12) mmol/µU/ml], CRP (I-5.5 ± 5.7, II-5.6 ± 5.7 mg/L), microalbuminuria [I-37.9 (9.2–47.8), II-17.85 (8.8–56.3) mg/24 h], PAI-1(I-6.4 ± 1.3, II-5.8 ± 1.8U/ml), homocysteine (I-11.6 ± 3.6, II- 12.7 ± 3.5 μmol/L) and FLI (I-5.29 (1.02–16.7), II-4,78 (1.62–20.69) (Table1). Statistical significance between groups I and II was obtained for the following parameters: systolic pressure (<0.001), diastolic pressure (*p* = 0.007), total cholesterol, LDL-cholesterol, triglycerides (<0.001), HDL-cholesterol (*p* = 0.003), mean insulin value in OGTT (<0.001), HOMA-IR (*p* = 0.003), uric acid (*p* = 0.013), homocysteine ​​(*p* = 0.044), siMS score (*p* = 0.005) (Table 1). On the basis of OGTT, glycoregulation disorder was determined in patients: overweight and obese patients without prediabetes (I-92.8, II-91.2%), IFG (I-2.4, II-1.4%), IGT (I-4.8, II-1.8%), DM2 (I-0, II-5.7%). The percentage of patients without prediabetes was calculated: overweight (I-34.8, II-38.0%), obese patients (I-65.2, II-62.0%). siMS score increased with age: I-3.03 ± 0.87, II-3.27 ± 0.90 (Table 1). siMS score in prediabetic patients IFG (I-3.83 ± 0.22, II-4.30 ± 1.75), IGT (I-3.31 ± 0.59, II-4.51 ± 1.7) was increased comparing to patients without prediabetes (I-2.99 ± 0,89, II-3.23 ± 0,82) and DM2 (II-3.41 ± 1.25) (Table 2).

**TABLE 1:** Anthropometrical and biochemical parameters - age group comparisons.

	Age group		
	≤30 (*n* = 167)	31+ (*n* = 284)	*p* value
Body weight (kg)	99.9 ± 22.9	95.5 ± 21.3	0.037^a^
BMI(kg/m^2^)	33.35 ± 7.07	33.18 ± 6.85	0.802^a^
Obese	112 (67.1%)	176 (62.0%)	0.277^b^
WC (cm)	104.0 ± 17.7	104.6 ± 15.4	0.671^a^
SBP (mmHg)	124.8 ± 14.9	130.9 ± 17.4	<0.001^a^
DBP (mmHg)	81.6 ± 10.5	84.7 ± 11.9	0.007^a^
Cholesterol (mmol/L)	5.1 ± 1.1	6.1 ± 1.2	<0.001^a^
HDL-chol. (mmol/L)	1.2 ± 0.4	1.3 ± 0.3	0.003^a^
LDL-chol. (mmol/L)	3.2 ± 0.9	3.8 ± 1.2	<0.001^a^
Triglycerides (mmol/L)	1.39 (1.05–2.05)	1.70 (1.24–2.35)	<0.001^c^
Glycaemia 0′ (mmol/L)	4.9 ± 0.7	5.2 ± 1.1	<0.001^a^
Glycaemia 30′	7.4 ± 1.5	8.2 ± 1.9	<0.001^a^
Glycaemia 120′	4.9 ± 1.3	5.4 ± 2.5	0.005^a^
Insulin 0′ (mlU/L)	22.6 (17.5–35.3)	18.3 (13.2–25.7)	<0.001^c^
Insulin 30′	86.5 (47.0–149.2)	60.3 (39.3–102.0)	0.001^c^
Insulin 120′	26.75 (17.30–67.90)	27.15 (17–51.05)	0.414^c^
Insulin average (mlU/L)	55.47 (31.57–88.07)	40.27 (26.50–60.53)	0.001^c^
HOMA IR	4.91 (3.54–7.55)	4.01 (2.99–6.12)	0.003^c^
HbA1c (%)	5.17 ± 0.48	5.60 ± 0.77	<0.001^a^
Glycoregulation impairment			
No	155 (92.8%)	258 (91.2%)	0.001^b^
IFG	4 (2.4%)	4 (1.4%)	
IGT	8 (4.8%)	5 (1.8%)	
DM	0	16 (5.7%)	
No glycoregulation impairement*			
And non-obese	54 (34.8%)	98 (38.0%)	0.521^d^
And obese	101 (65.2%)	160 (62.0)	
CRP (mg/dl)	4.4 (2.2–7.3)	3.5 (1.9–7.2)	0.423^c^
Urea (mmol/L)	4.3 ± 1.1	4.9 ± 1.3	<0.001^a^
Creatinine (μmol/L)	83.4 ± 23.5	80.9 ± 16.9	0.191^a^
Clarence creatinin (ml/min)	124.1 ± 50.0	113.3 ± 51.4	0.040^a^
Microalbuminuria (mg/24 h)	37.9 (9.2–47.8)	17.85 (8.80–56.3)	0.121^c^
ALT (U/L)	26 (18–40)	23 (18–32)	0.195^c^
AST (U/L)	21 (18–28)	21 (18–24)	0.096^c^
Gama-GT (U/L)	24 (16–33)	23 (16–32)	0.988^c^
Fatty liver index	5.29 (1.02–16.75)	4.78 (1.62–20.69)	0.265^c^
Uric acid (μmol/L)	337.0 ± 90.6	316.2 ± 82.4	0.013^a^
Homocysteine (μmol/L)	11.3 ± 3.6	12.7 ± 3.5	0.044^a^
PAI (U/ml)	6.4 ± 1.3	5.8 ± 1.8	0.140^a^
siMS score	3.03 ± 0.87	3.27 ± 0.90	0.005^a^

^a^ Independent Samples *t* test.

^b^ Fisher’s Exact test.

^c^ Mann-Whintey U test.

^d^ Pearson chi sqare test.

*Patients with glycoregulation impairment are excluded, therefore *n* = 412.

**TABLE 2:** Value of siMS score in different glycoregulation impairments and correlations with other parameters.

	Age group		
	Total	≤30 (*n* = 167)	31+ (*n* = 284)
Glycoregulation impairment			
No	3.14 ± 0.85	2.99 ± 0.89	3.23 ± 0.82
IFG	4.07 ± 1.19	3.83 ± 0.22	4.30 ± 1.75
IGT	3.77 ± 1.24	3.31 ± 0.59	4.51 ± 1.70
DM	3.41 ± 1.25	—	3.41 ± 1.25

siMS_score in correlation with	Age group		
	Total	<31	31+
Body weight	.320 (<0.001)	.382 (<0.001)	.310 (<0.001)
Body height	.028 (0.548)	−.008 (0.915)	.088 (0.139)
BMI	.342 (<0.001)	.434 (<0.001)	.294 (<0.001)
WC	.442 (<0.001)	.516 (<0.001)	.398 (<0.001)
Glycaemia 0′	.412 (<0.001)	.372 (<0.001)	.419 (<0.001)
HbA1c	.334 (<0.001)	.264 (0.001)	.326 (<0.001)
Insulin 0′	.335 (<0.001)^a^	.375 (<0.001)^a^	.388 (<0.001)^a^
Insulin average	.233 (<0.001)^a^	.327 (<0.001)^a^	.217 (<0.001)^a^
HOMA IR	.413 (<0.001)^a^	.421 (<0.001)^a^	.459 (<0.001)^a^
Systolic pressure	.356 (<0.001)	.419 (<0.001)	.303 (<0.001)
Diastolic pressure	.335 (<0.001)	.285 (<0.001)	.344 (<0.001)
Cholesterol	.231 (<0.001)	.287 (<0.001)	.151 (0.011)
HDL-cholesterol	−.583 (<0.001)	−.645 (<0.001)	−.595 (<0.001)
LDL-cholesterol	−.096 (0.043)	.079 (0.314)	−.233 (<0.001)
Triglycerides	.869 (<0.001)	.871 (<0.001)	.865 (<0.001)
ALT	.324 (<0.001)^a^	.375 (<0.001)^a^	.296 (<0.001)^a^
AST	.122 (0.011)^a^	.242 (<0.001)^a^	.052 (0.398)^a^
Gama-GT	.373 (<0.001)^a^	.468 (<0.001)^a^	.303 (<0.001)^a^
FLI	.656 (<0.001)^a^	.696 (<0.001)^a^	.614 (<0.001)^a^
CRP	.237 (<0.001)^a^	.336 (<0.001)^a^	.198 (0.005)^a^
Log CRP	.231 (<0.001)	.275 (0.001)	.210 (0.003)
PAI	.318 (0.012)	.121 (0.456)	.547 (0.010)
Microalbuminuria	.144 (0.005)^a^	.090 (285)^a^	.180 (0.006)^a^
Homocysteine	.231 (<0.001)	.166 (0.126)	.284 (0.076)
Uric acid	.285 (<0.001)	.288 (<0.001)	.319 (<0.001)
Creatinine	.117 (0.014)	.210 (0.007)	.063 (0.291)
Clarence creatinin	.099 (0.046)	.080 (0.328)	.138 (0.029)
Urea	.010 (0.835)	.024 (0.755)	−.043 (0.478)

All analyses are Pearson correlations except for Spearman correlation analysis.

^a^ Spearman correlation analysis.

**FIGURE 1:**
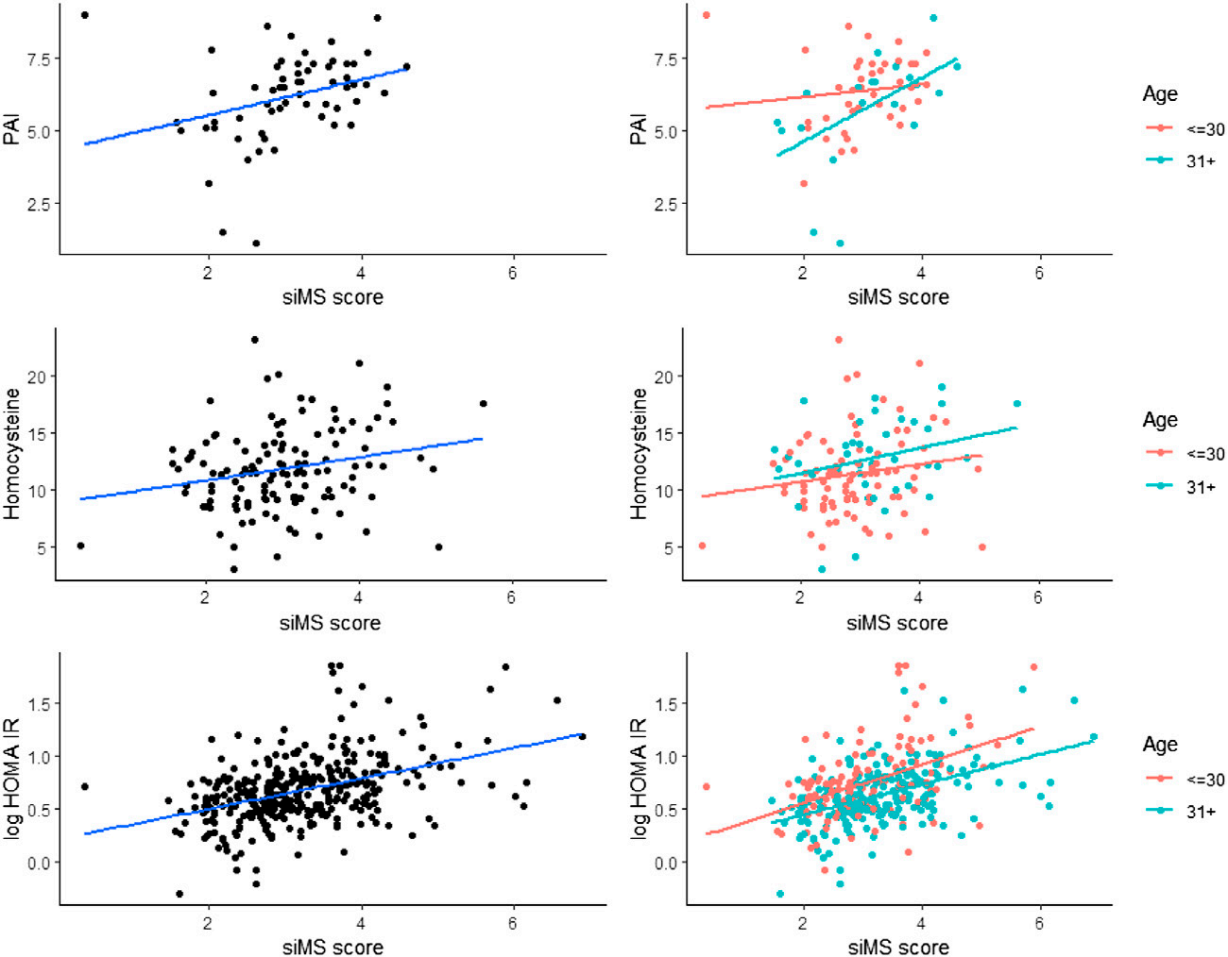
siMS score correlations with associated MS parameters: PAI-1, homocysteine and log HOMA IR.

**FIGURE 2:**
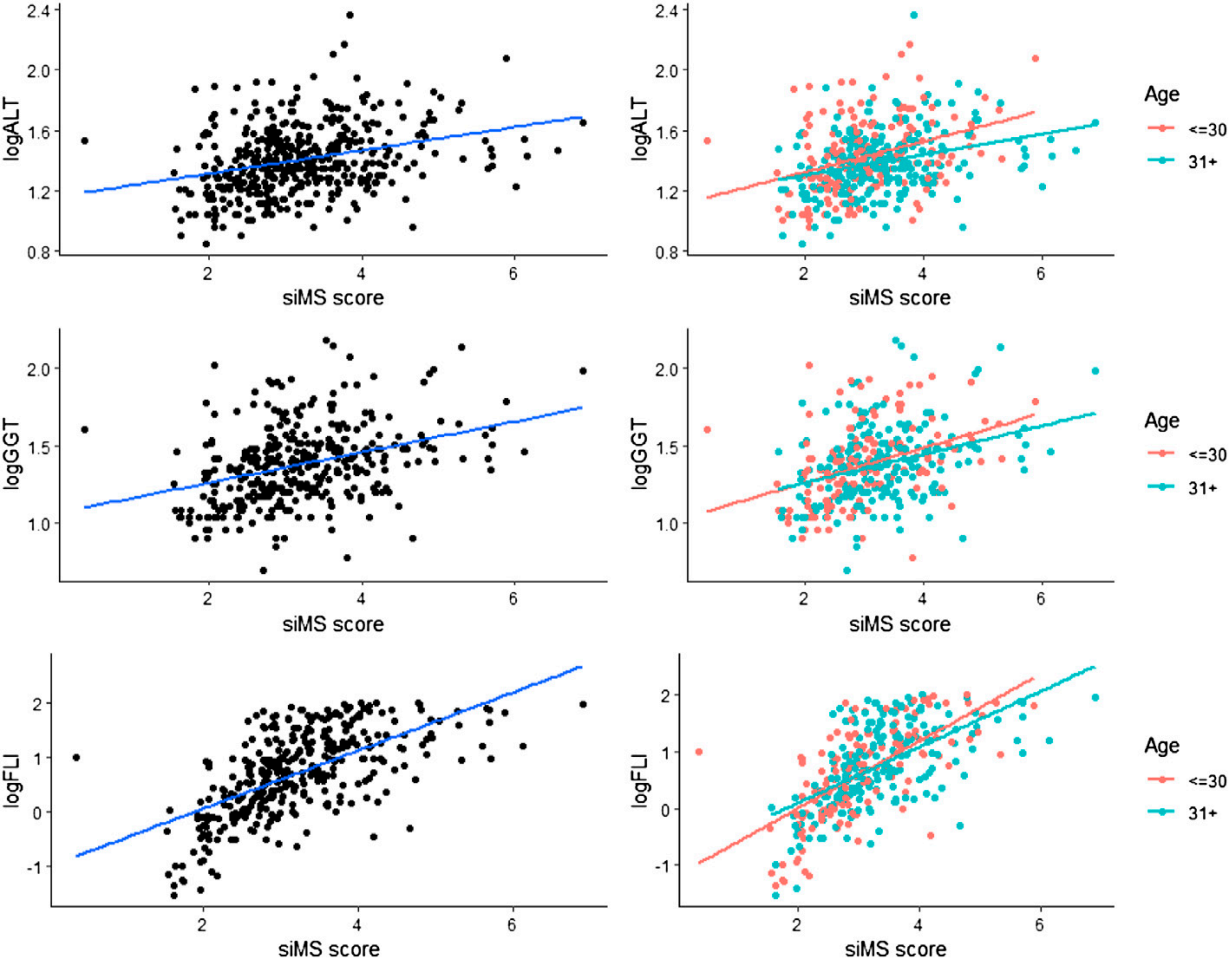
siMS score correlations with NAFLD parameters: log ALT, log GGT and log FLI.

Correlation of siMS score with anthropometric parameters body weight, BMI and long-term glycoregulation parameter HbA1C was obtained as well as with MS factors: WC, blood glucose, systolic and diastolic pressure, triglycerides and negative correlation with HDL-cholesterol (*p* < 0.01) in both groups. siMSscore correlated with associated factors of MS: hyperinsulinemia and IR (insulin, mean insulin value, HOMA-IR), ALT, GGT, FLI, uric acid (*p* < 0.01) in both groups as well as with CRP (*p* < 0.01) in group I, *p* = 0.005 in group II, log CRP (*p* < 0.01) in group I and *p* = 0.003 in group II. Correlations in II group: siMS score with PAI-1 (*p* = 0.01), microalbuminuria (*p* = 0.006), homocysteine ​​(*p* = 0.076) and creatinine clearance (*p* = 0.029) (Table 2). This study have found statistically significant correlation between siMS score and PAI-1, homocysteine, and log HOMA-IR seen in Figure 1. As well, there have been found significant correlation between siMS score and biochemical parameters for NAFLD (log ALT, log GGT, log FLI) (Figure 2).

## Discussion

Inflammatory and thrombosis factors secreted by adipose tissue lead to chronic inflammation, IR and the development of DM2 and cardiovascular disease (Hotamisligil et al., 1995). Our earlier research on children and adolescents showed the presence of MS in 37%, while the remaining 63% showed the presence of one or two MS criteria (mainly waist circumference and reduced HDL-cholesterol) and made up the group with pre-MS. Patients with MS were characterized by increased BMI, waist circumference, hyperinsulinemia, increased HOMA IR an IR parameter, hypertension, hypertriglyceridemia with low HDL-cholesterol, elevated PAI-1, decreased antioxidant protection and IGT in 18.1% patients. Patients with pre-MS had an elevated mean value of insulin in the OGTT, elevated HOMA IR, prothrombogenic status, microalbuminuria with reduced antioxidant protection. Elevated values ​​of HOMA IR and elevated mean insulin value in OGTT confirm increased insulin secretion, hyperinsulinism, reduced insulin sensitivity and insulin resistance in patients with MS and pre-MS already in young obese individuals (Dimitrijevic-Sreckovic, 2011). Positive correlation of PAI-1 with waist circumference in pre-MS, as well as positive correlation of PAI-1 with HOMA IR and glycaemia and negative correlation with HDL-cholesterol in children and adolescents with MS indicate that visceral fat tissue may be a connection between elevated PAI-1 values ​​and IR in patients with pre-MS and MS (Dimitrijevic-Sreckovic et al., 2007). Srećković B and colleagues showed that patients with MS had statistically significant higher values ​​of abdominal obesity, hypertriglyceridemia, hypertension, IR, CRP, microalbuminuria and homocysteine, which promote endothelial dysfunction and accelerated development of vascular complications.

On the other hand, hyperhomocysteinemia correlates with hyperinsulinism and IR and increases oxidative stress, which causes oxidative damage to the vascular endothelium, endothelial dysfunction, all of which contribute to hypertension and atherosclerosis. The correlation of homocysteine ​​with hyperlipoproteinemia, apolipoprotein B and elevated blood pressure indicates that homocysteine ​​can be a marker of the progression of arteriosclerosis (Sreckovic et al., 2017). The siMS score as a parameter for MS quantification has found significant application in our daily clinical work and helps us monitor how much after the application of a Mediterranean diet, physical activity and applied therapy there is a quantitative improvement in MS and thus a reduction in the risk of numerous chronic complications. In this study, the siMS score was higher in the elderly group compared to the group of young patients and increased with glycoregulation disorders. This finding is expected because over time with age the risk for overweight/obesity, hypertension, prediabetes/diabetes, hyperlipoproteinemia, which are the main factors for the diagnosis of MS, increases. Patients with prediabetes and diabetes had a higher siMS score compared to patients without diabetes precisely because of the presence of hyperglycemia as a causative factor of MS. Our research showed the expected correlation of the siMS score with the anthropometric parameters such as body weight, BMI, the long-term glycoregulation parameter HbA1C and with the MS factors: waist circumference, blood glucose, systolic and diastolic pressure, triglycerides, and a negative correlation with HDL-cholesterol in both groups of subjects with statistical significance. Correlation of siMS score with hyperinsulinemia and IR (insulin, mean insulin value, HOMA-IR) proves that hyperinsulinemia and IR underlie MS.

Hyperinsulinism and IR in the obese are responsible for hypertension and insulin acts on the reabsorption of sodium in the kidneys, increasing the circulating level and the development of hypertension (Ferrannini et al., 1987; DeFronzo and Ferrannini, 1991). In accordance with our results, and the research of Khoshhali et al. (2019) who point out that the siMS score has proven to be valid for detecting the risk of metabolic syndrome, it is simple and practical and can be used in clinical and research practice. Khan et al. (2020) analyzed the siMS score in people with and without MS and found that IR monitored *via* HOMA-IR was higher in people with MS compared to those without MS with statistical significance. Also, the siMS score was higher in subjects with MS (3.58 ± 0.725) compared to subjects without MS (2.83 ± 0.727). The area under the curve for the examined biochemical parameters was the highest for small dense LDL-cholesterol (sdLDL) and siMS score. The siMS score significantly correlated with anthropometric, biochemical, and hormonal risk factors and in the diagnosis of MS it showed a better performance than HOMA-IR, sdLDL, non-HDL, HbAlC and blood glucose. In our study, Figure 1. shows a significant correlation of the siMS score with logHOMA-IR, which is in agreement with the results of Khan et al. (2020), who showed that the siMS score as a method of mathematical assessment is highly correlated with IR, indicating the largest area under the curve to diagnose MS. The use of the siMS score can be useful both in diagnosis and as a useful tool for assessing the overall improvement or worsening of the disease in the prognostic monitoring of the patient, and some researchers point out that it can also be used as a biomarker for MS. Statistically significant correlation of siMS score with NAFLD parameters (ALT, GGT, FLI), uric acid, CRP, log CRP, in both groups and with PAI-1, microalbuminuria and homocysteine ​​in the elderly group proves that all these parameters are associated factors of MS. In agreement with the current results, our earlier research in obese patients with MS and without MS showed a positive correlation of the siMS score with CRP, uric acid, microalbuminuria, homocysteine, ​​and fibrinogen as associated factors of MS (Sreckovic et al., 2020). In another study we found that patients with MS had higher values of co-founding factors of MS (HOMA-IR, CRP, uric acid, ALT, GGT), this is confirmed by correlation with siMS score. siMS score further indicates that IR, CRP, fibrinogen, uric acid and NAFLD are co-founding factors of MS (Sreckovic et al., 2018). The results of this study show increased abdominal obesity, hypertension, hypertriglyceridemia, inflammatory factors, IR, homocysteine ​​and microalbuminuria as a marker of endothelial dysfunction already in patients with pre-MS as well as in patients with MS. Correlation of siMS score with creatinine in the young and creatinine clearance and microalbuminuria in the elderly indicates that initial disturbances of renal function may be associated factors of MS. Statistical significance between the young and elderly groups was obtained for systolic and diastolic pressure, total cholesterol, LDL-cholesterol, triglycerides, HDL-cholesterol, mean insulin value in OGTT, HOMA-IR, uric acid, homocysteine, siMS score, which indicates that the risk for atherosclerotic complications increases with age. The occurrence of prediabetes was also detected in young IFG (I-2.4, II-1.4%), IGT (I-4.8, II-1.8%), while DM2 was diagnosed in 5.7% of the older overweight and obese patients. Our earlier results showed that prediabetes was found in obese adolescents and young adults: elevated glycaemia was found in 3.7% of adolescents and 11.1% of 20–30-year-olds, while glucose intolerance was twice as common, 7.4% of adolescents and 22.2% of 20–30-year-olds (Dimitrijevic-Sreckovic and Vukasinovic, 2013). This data enhances importance of dietary and lifestyle intervention in children and adolescents, preventing those aforenamed conditions. Statistically significant correlation of siMS score with logALT, logGGT, log FLI shown in Figure 2 indicates that NAFLD is an associated factor of MS and siMS score can be a useful parameter for early detection of NAFLD. NAFLD is often associated with obesity and can manifest from steatosis, *via* steatohepatitis, fibrosis to liver cirrhosis and can often be associated with elevated serum homocysteine ​​levels (de Carvalho et al., 2013). This disorder was found in the youngest obese population, 7.3% in children aged 7–15, 18.9% in adolescents aged 16–20 and 29% in young people aged 20–30. Abdominal obesity associated with MS factors, hyperinsulinism, IR, inflammatory factors with oxidative stress are the main reason for NAFLD found in pre-MS 17.5% and in MS 29% (Sebekova and Sebek, 2018). Sebekova K. and Sebek J. showed that the siMS score can be useful to assess cardiometabolic risk in individuals not yet diagnosed with MS.

As discussed earlier, the main link between obesity, metabolic syndrome and NAFLD is inflammation, which induces IR. One of the pathologic hallmarks of obesity is macrophage infiltration of adipose tissue, that has been confirmed as a source of multipotent adult stem cells. Stem cell growth factor-beta (SCGF-β) shows activity on granulocyte-macrophages progenitor cells in combination with granulocyte-macrophage colony-stimulating factor (GM-CSF) and macrophage colony-stimulating factor (M-CSF). Recent study of Tarantino et al. (2020) documented that patients with more pronounced HOMA index had higher prevalence of moderate and severe steatosis compared to those with HOMA index below the median, as previously reported by Hui-Yun et al. (2013), although the median HOMA values overlapped according to gender. The fact that SCGF-β levels predicted only in males the severity of hepatic steatosis, could indicate that the adiposity of these subjects affects the inflammation status and/or the immune system.

According to this author, CRP and IL-6 levels could predict SCGF-β concentrations, but only in males. The mediating role of CRP is plausible if its functional role in inflammation is taking into account (Hui-Yun et al., 2013).

We used the siMS score in the analysis of people without MS because the percentage of these people in general population is not negligible. Their detection and implementation of preventive measures in lifestyle changes could slow down or stop metabolic disorders. The application of automated calculation of the siMS score using the electronic health record could help in the early detection of patients with cardiometabolic risk (Sebekova and Sebek, 2018). In our daily work to prevent diabetes, metabolic disorders, and atherosclerosis, we use individually adjusted Mediterranean menus, with different caloric intake, along with planned physical activity or walking for 1 h a day. A Mediterranean diet rich in monounsaturated fats from olive oil, omega 3-polyunsaturated fats from marine fish, complex carbohydrates and dietary fiber from fruits, vegetables, and cereals and poor in saturated fats of animal origin can contribute to the reduction of MS factors, abdominal obesity, hyperinsulinism, inflammatory and thrombosis factors, homocysteine ​​ and thus the prevention of diabetes and atherosclerosis. The effects of the Mediterranean diet on the prevention of diabetes and atherosclerosis (insulin resistance, hypertension, lipid status) have been shown, along with improving the antioxidant status and reducing homocysteine (Dimitrijevic-Sreckovic, 2011b).

## Conclusion

Abdominal obesity, hypertension, hypertriglyceridemia, inflammation factors, IR, uric acid, homocysteine and microalbuminuria as markers of endothelial dysfunction were increased in patients with pre-MS and MS. Correlation of siMS score with hyperinsulinemia IR (insulin basal, mean insulin value, HOMA-IR) confirmed that hyperinsulinism and IR are in the basis of MS. Statistically significant correlation of siMS score with NAFLD parameters, uric acid, CRP in both groups and with PAI-1, microalbuminuria and homocysteine ​​in the elderly group indicated that they are significant co-founding factors of MS. Patients with prediabetes and diabetes are characterized by more pronounced siMS score values compared to the patients without prediabetes precisely because of the presence of hyperglycemia as a causative factor of MS. Statistically significant correlation of siMS score with NAFLD parameters, logALT, logGGT and log FLI indicates that NAFLD is an associated factor of MS and siMS score can be a useful parameter for early detection of NAFLD. Correlation of siMS score with thrombosis factor PAI-1, endothelial dysfunction factor microalbuminuria, homocysteine as atherosclerosis marker, indicates higher risk for progression of endothelial dysfunction and atherosclerosis with age.

## Data Availability

The original contributions presented in the study are included in the article/Supplementary Material, further inquiries can be directed to the corresponding author.
